# Comparison of health-related quality-of-life measurement instruments in diabetic patients

**DOI:** 10.1080/13102818.2014.935572

**Published:** 2014-09-23

**Authors:** Stanislava Yordanova, Valentina Petkova, Guenka Petrova, Milen Dimitrov, Emilia Naseva, Maria Dimitrova, Elina Petkova

**Affiliations:** ^a^ Department of Social Pharmacy, Faculty of Pharmacy, Medical University of Sofia, Sofia, Bulgaria; ^b^ Department of Health Economics, Faculty of Public Health, Medical University of Sofia, Sofia, Bulgaria

**Keywords:** health-related quality of life, diabetes mellitus, EQ-5D, SF-6D, WHO-5

## Abstract

The objective of the study was to compare three different questionnaires (Short Form (SF)-6D, EuroQuol (EQ)-5D and WHO-5) to establish which one is more sensitive and which one gives an adequate assessment of the quality of life in patients with diabetes.

In an observational and transversal study with duration of 4 months, in 5 Bulgarian cities, 146 patients were randomly selected. The following quality-of-life measuring instruments were applied: 146 questionnaires SF-6D, 146 questionnaires EQ-5D and 103 questionnaires of WHO-5. Descriptive statistics, chi-Square and correlation coefficients were used for data analysis. The study assessed the quality of life of patients suffering from diabetes mellitus with a mean age of 57.39 years (standard deviation (SD) 17.087); 95% confidence interval (CI) 54.60–60.19; 76% of the patients had diabetes type 2. The patients received a mean SF-6D score of 0.6290, an EQ-5D score of 0.6272, a visual analogue scale score of 0.7158 and a WHO-5 score of 0.4635. Preferences measured by the SF-6D and by the EQ-5D showed significant correlations with one another, and the Pearson coefficient was *r* = 0.906 (*p* < 0.01). The most current version of SF-6D, based on the 2002 model, was found to be valid and reliable when compared to the EQ-5D and is a questionnaire alternative to assess preferences in economic analysis carried out in health care.

## Introduction

Every diabetic patient's life is unique. Many cannot effectively control their disease but all patients are unanimous in their opinion that diabetes has had a huge impact on their lives. They feel psychologically overwhelmed by the numerous rules that the disease requires them to follow. An added burden for them is the micro- and macro-vascular complications associated with both short-term and long-term diabetes management.

Therefore, assessing the quality of life (QoL) of patients is very difficult, due to the fact that each individual has their own subjective view on their physical, emotional and social well-being. This subjective opinion includes a cognitive element – satisfaction; as well as an emotional component – happiness. A declining QoL and depression can strongly influence a patient's commitment towards controlling their disease.[1–4]

There are different tools for measuring the QoL. A number of studies have analysed the link between QoL and different socio-economic factors such as national health insurance, additional health care services for diabetic patients, tailoring an individual treatment, changes to lifestyle, individual disease specifics (type of diabetes, duration), the presence of short-term or long-term complications, disabilities, psychological, social and demographic factors.[2,4,5] In studies comparing EuroQuol (EQ)-5D™ and Short Form (SF)-36®, the EQ-5D index is described as less sensitive towards differences in health status of patients as opposed to SF-36.[6–8] One study targeted at diabetic patients who have undergone a liver transplant surgery confirms that SF-6D® is less sensitive towards small QoL changes in these patients.[9]

Evaluating the QoL associated with health is particularly important for health care institutions; this includes general practitioners, pharmacists, nurses and is a vital component in identifying and establishing a suitable way of managing the disease, as well as increasing the overall QoL.

There is no direct approach in assessing the QoL. This is why item-measurement theory is applied when trying to evaluate QoL. It involves asking a category of questions, whose answers are translated into numerical values, after which they are input into statistical programs and QoL is evaluated.[10]

Jacobson et al. [11] with the help of generic and specific tools (Diabetes QoL (DQOL) and SF-36®) established that type-2 diabetic patients who do not undergo insulin treatment have reported an overall higher QoL than type-2 diabetic patients who are being treated with insulin. The latter, on their part, report a higher QoL than type-1 diabetic patients. When comparing EQ-5D™ and SF-36®, the latter shows a clearer differentiation between groups of patients and better sensitivity.[6–8]

Some tools are specific and are widely used for evaluation in diabetic patients. Their specificity comes from the tailoring for either type-1 or type-2 diabetes, whereas others evaluate both types at the same time, but they do offer the possibility of international comparison of the results. These so-called generic tools include the single-aspect EQ-5D, SF-6D, WHO-5; and the multi-aspect SF-36. Single-aspect measurements produce a summarized assessment for the QoL in an interval from 0 to 1, which represent poor and perfect health. Multi-aspect methods evaluate the QoL depending on different characteristics such as self-sufficiency, pain, motor function and others. They can generate an assessment within the values of 0–1 or 0–100, representing poor and perfect health, respectively.

A widely used generic tool for patients with diabetes is the Medical Outcomes Study Short-Form General Health Survey and all in all its varieties.[12,13]

It is due noting that, given the increasing pressure of limited treatment costs, most studies neglect the added benefits of additional services towards diabetic patients (which tend to improve patient satisfaction and QoL). The objective of the study was to compare the different questionnaires (SF-6D, EQ-5D and WHO-5) to establish which one is more sensitive and which one gives an adequate assessment of QoL in the respective group of individuals.

## Materials and methods

The study is an observational and transversal study that encompassed a 4-month period between May and August 2013.

### Quality-of-life instruments used in the study

Based on the literature, we selected four widely used QoL instruments and applied them to diabetic patients.

ShortForm-36D®. The SF-36D® questionnaire consists of 11 multi-dimensional questions divided in eight different subsections – overall 36 positions, which encompass: physical and social well-being, physical and emotional restraints, psychological health, vitality, pain and overall feeling of health and well-being. The structure of the questionnaire contains three main regions: functional status, well-being and overall health assessment.[14] The maximal number of points is 100 which in turn represents the best possible health condition and QoL. The answers to the questionnaire are tested for reliability and validity.[15]

ShortForm-6D®. The most recently created generic tool is the SF-6D [16] to extract given indicators from the extensive SF-36D. The SF-6D questionnaire presents a revised form of Sf-36D. It has six dimensions of health condition, whereby each dimension has from four to six possible levels of choice. This defines 18,000 different possible health conditions. The numerical borders for the evaluation lie from 0.30 to 1.00, where 1.00 is defined as ‘absolutely healthy’ (UK algorithm). Only a small amount of studies for this instrument exist with the expectation that its usage will increase in the future.

EuroQol-5D 3L™. In 1996 Brooks et al. created the instrument EQ-5D™ as an index for determining the quality-adjusted life years (QALYs). The main idea behind it was to establish a standardized evaluation system for describing the QALYs, which can then be used for international comparison of the populace's health status.[17]

EQ-5D 3L™ consists of five questions regarding mobility, independence, pain, everyday activities and psychological status. Each question has three possible answers. The maximum number of points that can be scored is 1, and the number indicates ‘absolute health’. An additional vision-analogue scale from 0 to 100 has been developed where 100 represents the best possible health condition.

WHO-5 index for QoL (1999 version). The WHO-5 index for QoL is derived from a scale with more indicators. It was developed from a WHO project for diabetic patients’ QoL.[18] During the first psychometric assessment 10 aspects were selected out of the 28 original ones because of the uniformity they showed in the European countries that took part in the study.[19] Since only positively formulated indicators can give a real evaluation for the psychological status, the aforementioned 10 aspects were reduced to 5 (WHO-5). These five include good mood (good mood, calmness), vitality (being able to wake up refreshed and feeling rested and energized) and interests (if some activity has caused an interest). The result is interpreted after combining the points acquired from the five answers, where 0 is the lowest possible result and 25 is the highest possible QoL. To produce a percentile result, the final points are multiplied by 4. The percentile result is used to establish a change in the QoL, where a 10% alteration is a significant change.

### Patient selection

A group of 146 people were randomly selected to participate in the study. All 146 participants were diabetic patients between the ages of 18 and 91 with the average age being 57.39 years (with a standard deviation (SD) of 17.087 and 95% confidence interval (CI) of 54.60–60.19). The interviewed patients were from the capital, Sofia, and from four other big cities – Burgas, Varna, Plovdiv and Shumen. They were recruited in the endocrinology offices. Every first patient on the day of observation at the office who agreed to participate was included in the sample. Each patient was interviewed by experienced pharmacists.

The study sample contained predominantly female patients, while the male patients comprised approximately 30%. Of all the patients in the study, 76% had type-2 diabetes.

More than half of them were being treated with oral anti-diabetes therapy; 73% had developed complications due to the diabetes. Parts of the chronic complications were polyneuropathy, retinopathy, nephropathy and kidney failure, dermopathy as well as diabetes foot and following amputations.

Patients were interviewed with each one of the QoL instruments at a time convenient for them and the answers were processed statistically.

### Materials

During the course of the study the following instruments were used to measure the QoL in diabetic patients and the following materials were obtained: 146 standard SF-6D questionnaires for evaluation of health-related QoL; 146 standard EQ-5D 3L questionnaires for evaluation of health-related QoL; and 103 standard WHO-5 questionnaires for indexing the QoL (1999 version).

The results obtained through these different questionnaires were then compared to establish which one was more sensitive and which one gives an adequate assessment of QoL in the respective group of individuals. The results were processed statistically through SPSS v.16.

### Statistical methods

Descriptive statistical methods were used when interpreting the questionnaires for evaluation of the QoL. The interviewed people were observed in the following categories: sex, type of therapy, existing complications and type of questionnaire.

The following statistical tests were used to establish if there was a statistically significant difference in the QoL: ANOVA; Mann–Whitney U test; Kruskal–Wallis test; chi-square. A correlation analysis was also applied to measure the strength of the correlation between different indexes (Pearson correlation coefficient).

## Results and discussion

**Figure 1.**
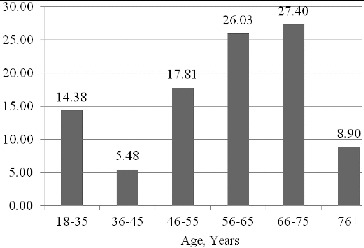
Distribution of the patients by age groups (%).

We separated the patients into six groups based on age. The highest per cent of patients were in the retirement age (between 66 and 75 years) and immediately after them were patients in pre-retirement age (between 56 and 65 years). The percentage of patients in the working ages of 46–55 years was approximately 20% (Figure 1).

The highest sample sizes were in the cities of Sofia (28.1%) and Shumen (30%). Thirteen per cent of the observed patients were with type-1 diabetes, 76% with type 2 and 11% with other types of diabetes. The patients who fell into the ‘other types’ category were those with maturity onset in diabetes of the young and latent autoimmune diabetes of adults and one pregnant patient with gestational diabetes.

The percentage of patients with complications was traced. In the cohort, 73% had some sort of complication associated with the disease against 27% without complications.

**Figure 2.**
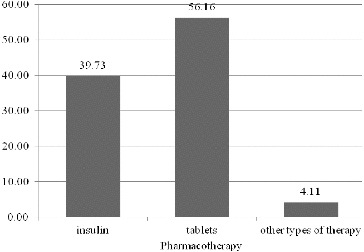
Distribution of the patients according to their pharmacotherapy (%).

Good control over the disease involves having an adequate therapy. In the sample of analysed patients, 56% were on oral anti-diabetes therapy and 40% of the patients were on an insulin-based therapy. The remaining 4% were on ‘other types of therapy’, i.e. on a combined therapy of insulin and oral anti-diabetes therapy, therapy with new groups of medicinal products (DPP-4, GLP-1) (Figure 2).

**Figure 3.**
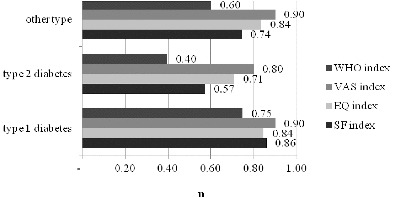
Quality-of-life indexes measured with SF-6D, EQ-5D and WHO-5 questionnaires.

As mentioned above, EQ-5D is one of the most frequently used tools for evaluating the QoL as it gives a summarized assessment within the interval 0–1 and can be used in the cost–utility analysis for the generation of QALY. The mean QoL among the interviewed patients (using EQ-5D) varied between 0.84 and 0.71. The lowest self-assessment value was obtained in patients with type-2 diabetes. Overall, the values obtained from all three groups of diabetes patients could be classified as low (Figure 3).

The visual analogue scale (VAS) is an addition to EQ-5D and is based on a self-assessment of QoL. Using this method, the interviewed patients gave their own opinion of QoL in regard to EQ-5D.

The third method used to measure QoL was the SF-6D questionnaire. It falls under the single-aspect tools. The results obtained showed lower QoL compared to that evaluated on the basis of VAS and an almost identical result to that from EQ-5D.

The WHO-5 questionnaire is probably characterized with the lowest values, as it measures four health conditions and combines them into a single value.

The patients showed a higher value for QoL in the VAS for the EQ-5D questionnaire in comparison to the calculated index value for QoL. Apart from that, in all four methods used the lowest values were observed in patients with type-2 diabetes, followed by patients with ‘other types’ of diabetes and highest values were observed in type-1 diabetic patients. The reasons for these differences could be due to the age of the interviewed patients, the presence of complications and other characteristics. This drew our attention towards clarifying these questions, as well as assessing the statistical significance of the presented differences.

**Figure 4.**
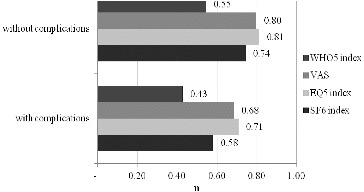
Differences in health-related QoL in diabetic patients with or without complications.

Figure 4 shows the differences in QoL in diabetic patients with complications and without complications. Logically, as expected, in all four groups, the patients with complications showed a poorer QoL. The results obtained were also lowest for the WHO-5 questionnaire, followed by SF-6D and, again, the values were identical for VAS and EQ-5D.

The next indicator we followed was the statistical significance of the differences between the mean values of QoL measured by the different tools (Table 1). The observed statistically significant differences for QoL of patients depended on the type of diabetes and the presence of complications due to the disease. The city of residence, sex and therapy showed no statistically significant influence over the QoL of the patients.

**Table 1.** Assessment of health-related quality of life in 146 diabetic patients with SF-6D, EQ-5D, VAS, WHO-5 questionnaires.

	VAS	SF6 index*	EQ index	WHO5_IQL*
Sex	0.468**	0.970	0.940**	0.968
Type diabetes	<0.001***	<0.001	<0.001***	<0.001
City	0.359***	0.429	0.245***	0.295
Therapy	0.043***	0.212	0.865***	0.866
Complications	0.007**	0.003	0.031**	0.032

*ANOVA; **Mann–Whitney U test; ***Kruskal–Wallis test.

An additional point of interest for the analysis was whether or not there was a difference between the characteristics of QoL included in the methods used (Table 2). The highest average value was for the component ‘social contacts’ from the SF-6D scale, and the lowest one, for ‘daily activities’. Among the EQ-5D components the mean value for ‘independence’ was the highest and the lowest one was for ‘uneasiness/repression’. From the WHO scale, the highest mean values fell under ‘rested’ and the lowest ones under ‘happy, good mood’. These results confirm that diabetes as a disease lowers the QoL, deepens the feeling of depression and lowers a person's joy of life. These results can be used by health care professionals as critical indicators that have a huge impact on patients and which they should take care to change.

**Table 2.** Preferences of the questionnaires and summarized performance limits.

Measure preference	*N*	Mean	SD	Min	Max
SF6 physical activities	146	70.09	28.292	17	100
SF6 everyday activities	146	61.13	32.301	25	100
SF6 social contacts	146	77.53	28.949	20	100
SF6 pain	146	68.61	27.786	17	100
SF6 psychological condition	146	65.34	25.547	20	100
SF6 vitality	146	65.75	26.880	20	100
EQ5 mobility	146	74.66	21.192	33	100
EQ5 self-care	146	89.04	21.122	33	100
EQ5 ordinary daily activities	146	76.71	21.574	33	100
EQ5 pain/discomfort	146	71.00	23.575	33	100
EQ5 anxiety/depression	146	65.75	22.470	33	100
WHO happy, in good mood	103	51.46	23.926	17	100
WHO calm	103	62.30	24.361	17	100
WHO vitality/activity	103	64.40	27.421	17	100
WHO fresh, rested	103	65.70	24.234	17	100
WHO interesting everyday	103	36.89	19.337	17	83
VAS	146	71.58	22.056	10	100

Note: 0 = the worst possible health state, 100 = the best possible health state.

The patients were with a mean SF-6D score of 0.6290, EQ-5D score of 0.6272, VAS score of 0.7158 and WHO-5 score of 0.4635. Of the four scales, the highest mean for QoL was measured by the VAS for the EQ-5D questionnaire, whereas the lowest numerical value was obtained from the WHO questionnaire (Table 3).

**Table 3.** The average values of the estimated quality of life, as measured by the generic instruments SF-6D, EQ-5D, WHO-5D and VAS in patients with diabetes.

Measure preference	*N*	Mean	Std. deviation	Minimum	Maximum
VAS	146	0.7158	0.22056	0.10	1.00
SF-6D	146	0.6290	0.28972	0.00	1.00
EQ-5D	146	0.6272	0.31450	−0.11	1.00
WHO-5D	103	0.4635	0.25731	0.04	0.92

The four measuring preferences showed significant correlations with one another, where the strongest relation was between EQ-5D and SF-6D, and the Pearson coefficient was 0.906 (*p* < 0.01). The lowest correlation was between WHO-5D and VAS (*r* = 0.746) (Table 4).

**Table 4.** Linear correlation between the selected generic instruments to assess quality of life: SF-6D, EQ-5D, WHO and VAS.

	VAS	SF-6D	EQ-5D	WHO-5D
VAS	1	0.880**	0.811**	0.746**
SF-6D	0.880**	1	0.906**	0.842**
EQ-5D	0.811**	0.906**	1	0.780**
WHO-5D	0.746**	0.842**	0.780**	1

**p < 0.01.

Transitional analyses showed that the single-aspect generic measurers for QoL (EQ-5D, SF-6D, WHO-5D and VAS) can be used for evaluation of health care to analyse the impact they have on the QoL of diabetic patients.

## Conclusions

Comparison and frequency analysis of the methods used to evaluate the QoL associated with diabetes showed that out of the four scales, the highest average was the mean value of QoL measured by the VAS for EQ-5D and the lowest one was calculated and obtained through the WHO questionnaire. The four measuring methods were found to be strongly inter-connected, with strongest relation between the indexes of EQ-5D and SF-6D and a relatively weaker one between WHO-5D and VAS. The most current version of SF-6D, based on the 2002 model, was found to be valid and reliable when compared to the EQ-5D and is a questionnaire alternative to assess preferences in economic analysis carried out in health care.
